# A Preliminary Examination of Nicotine-Free Electronic Cigarette Use During Cessation From Combustible Cigarettes

**DOI:** 10.3389/fpsyt.2019.00559

**Published:** 2019-08-08

**Authors:** Alyssa L. Peechatka, Elena K. Molokotos, Maya Zegel, Scott E. Lukas, Amy C. Janes

**Affiliations:** ^1^Functional Integration of Addiction Research Laboratory (FIARL), McLean Imaging Center, McLean Hospital, Belmont, MA, United States; ^2^Harvard Medical School, Boston, MA, United States; ^3^Department of Psychology, Suffolk University, Boston, MA, United States; ^4^Behavioral Psychopharmacology Research Laboratory (BPRL), McLean Imaging Center, McLean Hospital, Belmont, MA, United States

**Keywords:** tobacco smoking, electronic cigarettes, nicotine replacement therapy, smoking cue reactivity, extinction

## Abstract

Despite the availability of smoking cessation strategies, smoking cue-induced craving remains a relatively untreated relapse risk factor. Utilizing nicotine-free electronic cigarettes (e-cigarettes) to extinguish the motivational influence of smoking cues may be a viable approach to address cue reactivity. In this pilot study, 26 daily tobacco smokers used nicotine-free e-cigarettes while being maintained on daily transdermal sustained-release nicotine replacement therapy (NRT) to mitigate pharmacological withdrawal. Sensitivity to cue-induced craving, measured by the rise in craving after a visual cue exposure task, was assessed at a baseline visit after smoking as usual and again after 2 weeks of nicotine-free e-cigarette and NRT use. Participants’ pattern and amount of tobacco cigarette smoking were evaluated on both visits and 1 month posttreatment. Cue-induced craving significantly decreased after the 2-week intervention, yet withdrawal scores increased during this time. One month after study completion, participants continued to report significantly lower overall cigarette craving and conventional tobacco cigarette use. Including the 34.8% that were totally abstinent, 65.2% reported smoking fewer than 10 cigarettes per week (compared to 87.2 per week at baseline for the entire group). A linear regression revealed that greater baseline cue-induced craving predicted better outcomes, whereas more withdrawal at the e-cigarette visit was related to more smoking at 1 month. This proof-of-concept pilot study suggests that the addition of *ad libitum* nicotine-free e-cigarettes to an existing strategy of transdermal NRT may attenuate cue-induced craving for tobacco smoking. A larger sample that is powered for detecting additional factors and longer-term outcomes is warranted.

## Introduction

Even when using available smoking cessation aids, relapse rates to smoking remain high (1). To address this limitation, there is the strong need to define treatments targeting relapse-precipitating factors, which are relatively unmitigated by standard pharmacotherapy. For instance, although symptoms of nicotine withdrawal can be attenuated by nicotine replacement therapy (NRT) and other pharmacotherapies, such as varenicline [for review, see Ref. (2)], smoking cue-induced craving is particularly difficult to mitigate, causing relapse months to years after cessation (3, 4). Although evidence strongly indicates that transdermal NRT does not attenuate cue reactivity (3–5), other studies suggest that medications, such as varenicline (6), and shorter-acting NRT, such as nicotine gum/lozenge (7), blunt cue reactivity. However, such medications are not consistently effective at reducing cue reactivity (8), and enhanced cue reactivity predicts relapse even when combined transdermal and short-acting NRT are used (9).

Collectively, this suggests the need for additional methods aimed at reducing cue reactivity, which potentially could be used in conjunction with currently available pharmacotherapy. Extinction is one potential behavioral method, where smoking cues are decoupled from acute nicotine administration, resulting in the devaluation of the smoking cues’ motivational influence. Extinction therapies, such as cue exposure therapy (10), have been historically ineffective (11) partly because of context-driven renewal (12). Such renewal occurs when extinction takes place in a laboratory or clinical setting and does not effectively translate to real-world settings, leading to a “renewal” of cue reactivity in these contexts. Thus, there is the need to extinguish cues across real-life environments where individuals regularly smoke and encounter smoking-related stimuli.

It is plausible that cue reactivity may be reduced by replacing daily use of conventional tobacco cigarettes with non-nicotine-containing e-cigarettes when use occurs in environments where individuals typically smoke. In the short term, nicotine-free e-cigarette use may prevent relapse to smoking tobacco cigarettes by allowing the individual to continue to engage in behavior similar to smoking, without the reinforcing effects of nicotine, leading to a devaluation of smoking cues after repeated use. A limitation of using nicotine-free e-cigarettes alone is that individuals may still experience withdrawal symptoms. Adjunctive use of transdermal NRT may mitigate this issue because transdermal NRT provides a steady dose of nicotine to avert withdrawal symptoms without the reinforcing effects of dopamine bursts typically observed when nicotine is inhaled (13). The combination of non-nicotine e-cigarettes with transdermal NRT ensures that nicotine withdrawal and cue reactivity are simultaneously addressed. Although no other study to date has evaluated this combined approach, we designed the present proof-of-concept pilot study to test our theory that combining nicotine-free e-cigarettes with transdermal NRT is a viable strategy to allow individuals to engage in cue extinction without experiencing symptoms of acute withdrawal. Overall, this pilot study is the first step in assessing the potential utility of this combined treatment approach as a cessation tool that is specifically targeting cue reactivity.

## Methods

### Participants

Twenty-six participants (eight females) who expressed a desire to quit smoking tobacco cigarettes were recruited between January 2015 and July 2018. Participants had to be between 18 and 45 years old, report smoking traditional cigarettes daily for at least the past 6 months, and be nicotine dependent, as measured by the Fagerstrom test for nicotine dependence (FTND) (14). Average participant characteristics are found in **Table 1**. Participants also had to express a willingness to transition from their regular cigarette use to the provided transdermal NRT and nicotine-free e-cigarettes. However, participants could not be currently using NRT or e-cigarettes and could only report infrequent use (<1× per month) of other forms of nicotine (cigar, pipe, chewing tobacco, etc.).

**Table 1:** Represents participant characteristics as measured on the baseline visit. Data represent the mean and standard deviation (SD), except in the case of sex, which is noted as percent of total population and n.

	n = 26
*Sex, % (n)*	
Females	30.8 (8)
Males	69.2 (18)
*Age*	27.7 (5.7)
*Years of education*	15.1 (1.5)
*Cigarettes per day*	12.5 (6.1)
*Expired CO*	16.8 (12.1)
*Pack-years*	7.3 (7.0)
*FTND scores*	4.5 (1.8)
Total Craving Score*	17.7 (6.7)

*Tiffany Questionnaire of Smoking Urges.

Participants were excluded for current illicit drug or alcohol dependence, major depressive disorder within the past 6 months, and current or lifetime history of schizophrenia, schizoaffective disorder, bipolar disorder, or psychotic disorders not otherwise specified [confirmed *via* Structured Clinical Interview for DSM-IV Axsis I disorders: Text Revision (SCID-IV-TR)]. Exclusionary criteria also included current serious medical illness, pregnancy, and recent drug/alcohol use (confirmed by the QuickTox11 Panel Drug Test Card, Branan Medical Corporation, Irvine, California; Alco-Sensor IV, Intoximeters Inc., St. Louis, MO). All procedures were completed at McLean Hospital, and the protocol was approved by the Partners Human Research Committee. Participants provided both verbal and written informed consent after receiving a complete description of the study.

### Procedures

The overall study included four study visits: 1) **baseline visit**, where precessation metrics were collected, 2) A brief **check-in visit** 1 week after the baseline visit to assess compliance, 3) An **e-cigarette visit** 2 weeks after the baseline visit after the transition to transdermal NRT and nicotine-free e-cigarette use, and 4) a **follow-up visit** approximately 1 month after the e-cigarette visit to assess smoking behavior. The baseline and e-cigarette visits followed the same general timeline. The 2-week duration between the baseline and e-cigarette visits was chosen to allow for the transition to e-cigarettes. This duration also was chosen to prevent potential habituation to the cue reactivity task. Previous work has shown that cue exposure repeatedly evokes craving even with a shorter, 1-week duration between cue presentations (15).

Before the baseline study visit, participants were instructed to smoke as usual. Expired carbon monoxide (CO) was measured at the beginning of each study visit to provide a biochemical measure of smoking behavior. To standardize the duration between the last cigarette smoked and all procedures, participants smoked one of their own cigarettes (baseline visit) approximately 1 h before the cue exposure task. Craving was evaluated before and after cue exposure. After cue exposure, withdrawal was measured by the Wisconsin Smoking Withdrawal Scale [WSWS (16)] (∼3.5 h after smoking).

At the end of the baseline visit, participants were provided transdermal NRT adjusted to their reported quantity of cigarette use (14 mg or 21 mg) and instructed in how to use this product. Participants also received nicotine-free e-cigarettes. The Apollo Challenger brand e-cigarettes (https://www.apolloecigs.com/en/e-cigarette-vape-kits/cigalikes-disposables/apollo-challenger-kit) were used, which are cigarette-like in appearance and were used in conjunction with the tobacco or menthol flavored e-liquid (matched to the participant’s typical use) containing 0 mg nicotine. Participants were instructed to discontinue using their tobacco cigarettes at this time and were only to use the provided e-cigarettes, but they could use them as frequently as they liked. Participants were told that the amount of nicotine in both the e-cigarette and NRT combined was roughly equivalent to the amount they received in their typical smoking pattern, but that there was the possibility that the e-cigarette could contain no-nicotine. All participants received 0 mg e-cigarettes and were debriefed at the end of the study. To equate the study day timelines on the baseline and e-cigarette visits, participants used one of their e-cigarettes for 15 min approximately 1 h before the cue reactivity task. Between the baseline and e-cigarette visits, participants were asked to fill out daily diaries to document their use of e-cigarettes, NRT, and tobacco cigarette.

### Measures

During the baseline and e-cigarette visits, subjective cigarette craving was measured using the Brief Questionnaire of Smoking Urges (QSU; 17) before (QSU_pre_) and after (QSU_post_) cue exposure. Change scores representing sensitivity to cue-induced craving were calculated by subtracting QSU_pre_ from QSU_post_; thus, positive values represent enhanced cigarette craving after cue exposure. During the 1-month follow-up visit, the QSU was administered once without cue exposure. The QSU has a factor structure consisting of two factors: factor 1 is associated with reward and urge aspects of craving, whereas factor 2 is associated with mitigating symptoms of withdrawal (17). Both factors were considered in the subsequent analyses.

### Cue Exposure Task

To measure sensitivity to cue-induced craving, participants completed a five-run, 26.5-min visual cue exposure task using the exact same protocol and validated images (visual cues) that we have used in our prior studies (18, 19). During this task, participants were shown 50 smoking, 50 neutral, and 10 target images in a pseudorandom order (only two images of the same type were shown in a row). This task was chosen because we have previously shown that it increases subjective craving after exposure to the cues (19). Participants were instructed to attend to all images and respond with a button press to the target images. Smoking images included smoking-related content, such as people smoking, people holding cigarettes, or cigarettes alone. Neutral images were matched for content in that they involved people, hands, or objects, such as pens or paintbrushes. Target images were animals, and participants were asked to press a button upon seeing a target image. This manipulation was included to ensure that participants attended to the task. Images were comparable but novel at each visit. A baseline manipulation check comparing craving (QSU factor 1) scores before and after cue exposure confirmed a rise in craving after task completion (*t*(25) = -2.48, *p* = 0.020).

## Results

### Smoking Behavior

**Figure 1:**
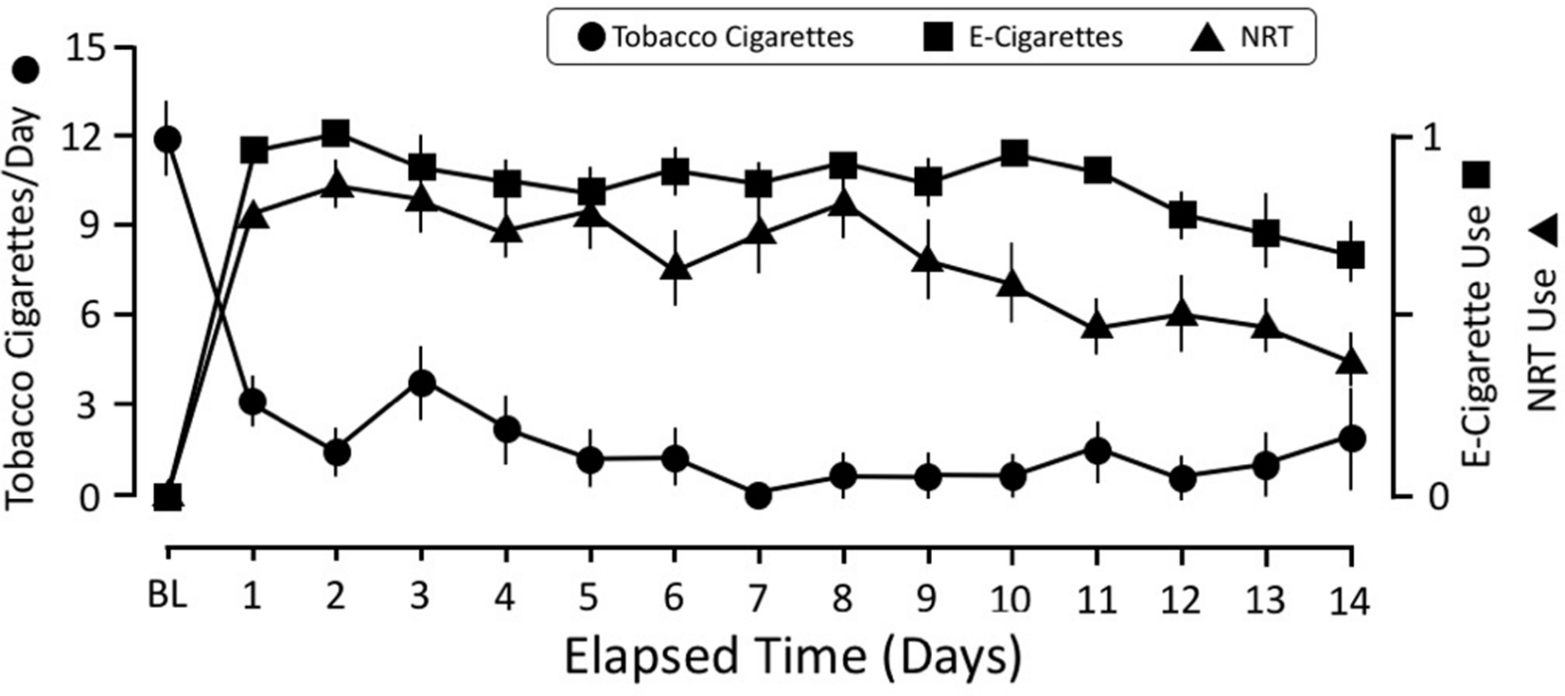
Time course of smoking behavior, NRT, and e-cigarette use over the 2-week study. The scale for self-reported number of tobacco cigarettes is the average number of cigarettes per day. The scale for NRT and e-cigarettes was coded for either used or did not use that day. This strategy was adopted because it was difficult for participants to equate their e-cigarette use with number of “cigarettes.” The e-cigarette data were coded as “1” if they reported using an e-cigarette that day and a “0” if they did not use it. The same strategy was used for tracking NRT use: a “1” indicated that they wore the patch that day, whereas a “0” indicated that they did not wear it. NRT, nicotine replacement therapy.

**Figure 2:**
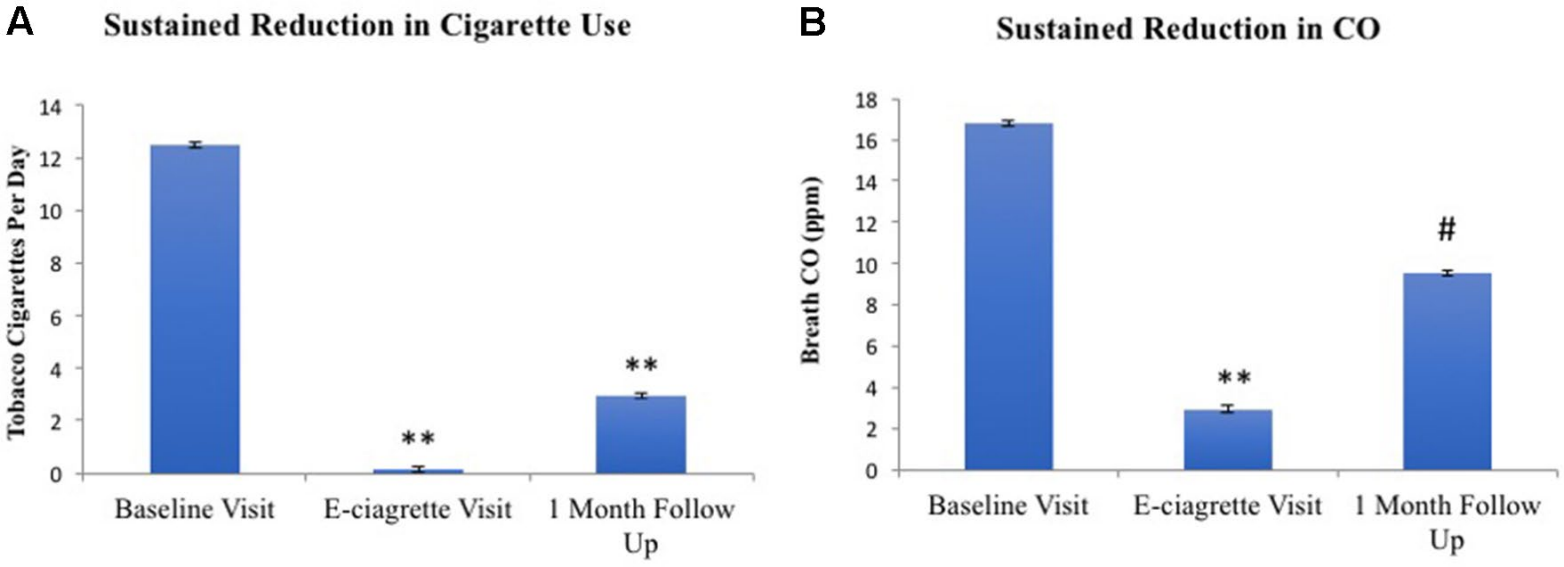
Tobacco smoking **(A)** and CO **(B)** at all three study visits. Relative to the baseline visit, reduced levels of self-reported smoking and CO levels were noted on the e-cigarette and 1-month follow-up visits. **p< 0.001, ^#^ *p* = 0.053.

All 26 participants completed both the baseline and e-cigarette study visits. Daily diary self-report (N = 24; two participants did not complete this aspect) demonstrated that tobacco smoking was reduced on the first day that transdermal NRT and e-cigarettes were provided and remained below three cigarettes/day for the remainder of the 2-week period (**Figure 1**). Overall, participants used NRT on 66.5% of days and e-cigarettes on 87.3% of days while enrolled in the study, which was reflected by the rapid increase in their use on day 1 of the study after the baseline study visit. However, use of the NRT started to decline by the eighth to ninth day of the study and ended at 40% used on day 14. The reduction in daily tobacco cigarette use between the baseline (*M* *_1_* = 12.46) and e-cigarette visits [*M* *_2_* = 0.16; *t*(23) = 9.95, *p* < 0.001] was statistically significant (**Figure 2A**). This reduction in smoking was confirmed by significantly lower expired CO on the e-cigarette (*M* *_2_* = 2.92) visit relative to the baseline visit [*M* *_1_* = 16.81; *t*(25) = 5.47, *p* < 0.001; **Figure 2B**]. There was a significant rise in withdrawal measured by the WSWS from the baseline to e-cigarette visits [*M* *_1_* = 42.8; *M* *_2_* = 49.4, *t*(25) = 2.3, *p* = 0.028; data not shown]. No other form of nicotine use was reported during this period.

### Changes in Cue-Induced Craving

**Figure 3:**
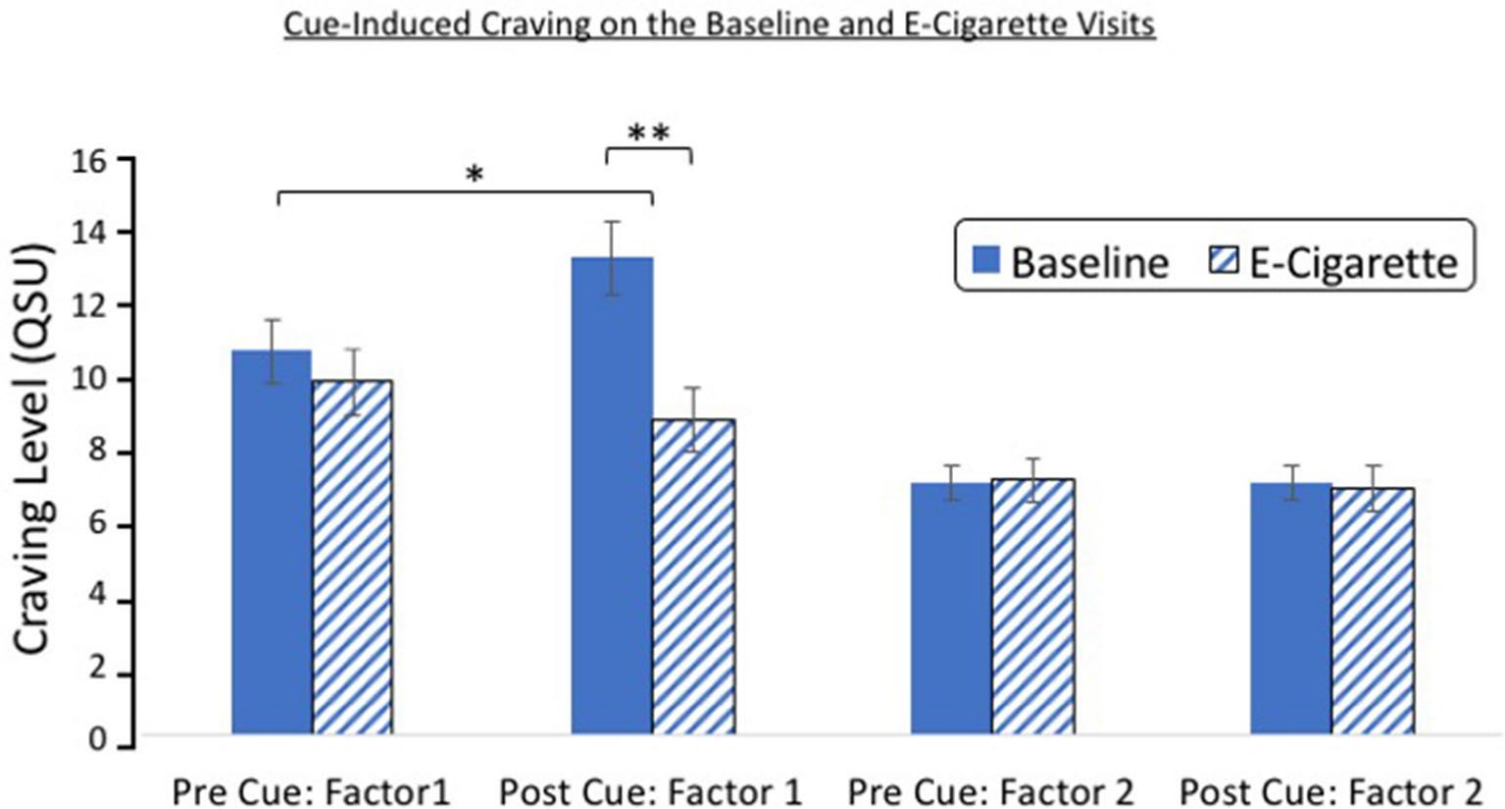
Cue reactivity on the baseline and e-cigarette test day. A significant interaction was found between study visit (baseline/e-cigarette), cue exposure (pre/post), and QSU factor (factor 1/factor 2). Follow-up analysis indicated that this effect was driven by factor 1, which showed an increase after cue exposure on the baseline visit but not on the e-cigarette visit. Post-cue craving was greater on the baseline relative to the e-cigarette visit for factor 1. *p = 0.02, **p < 0.001. CO,carbon monoxide.

On average, 15.32 ± 2.7 days separated the baseline and e-cigarette visits. A repeated-measures ANOVA of visit (baseline/e-cigarette), cue exposure (pre/post), and QSU factor (factor 1/factor 2) revealed that there was a significant three-way interaction [*F*(1,24) = 11.56, *p* = 0.002; **Figure 3**]. Significant interactions were also found between visit and factor [*F*(1,24) = 26.63, *p* < 0.001] and visit and cue exposure [*F*(1,24) = 5.08, *p* = 0.03]. A main effect of factor also was noted [*F*(1,24) = 64.88, *p* < 0.001]. Follow-up t-tests indicate that these findings were driven by factor 1 of the QSU. Although factor 1 was significantly increased after cue exposure at baseline [*t*(25) = 2.48, *p* = 0.02], this rise was not observed during the e-cigarette visit [*t*(24) = 1.36, *p* = 0.19]. Factor 2 did not change after cue exposure on either visit. After cue exposure, factor 1 was greater during the baseline visit relative to the e-cigarette visit [*t*(24) = 4.80, *p* < 0.001], whereas no such effect was found for factor 2 [*t*(24) = 0.15, *p* = 0.88]. Finally, the elevation in craving after cue exposure (QSU_post_-QSU_pre_) was greater for factor 1 on the baseline relative to the e-cigarette visit [*t*(24) = 2.86, *p* < 0.001] but not for factor 2 [*t*(24) 0.087, *p* = 0.93].

### One-Month Follow-Up

**Figure 4:**
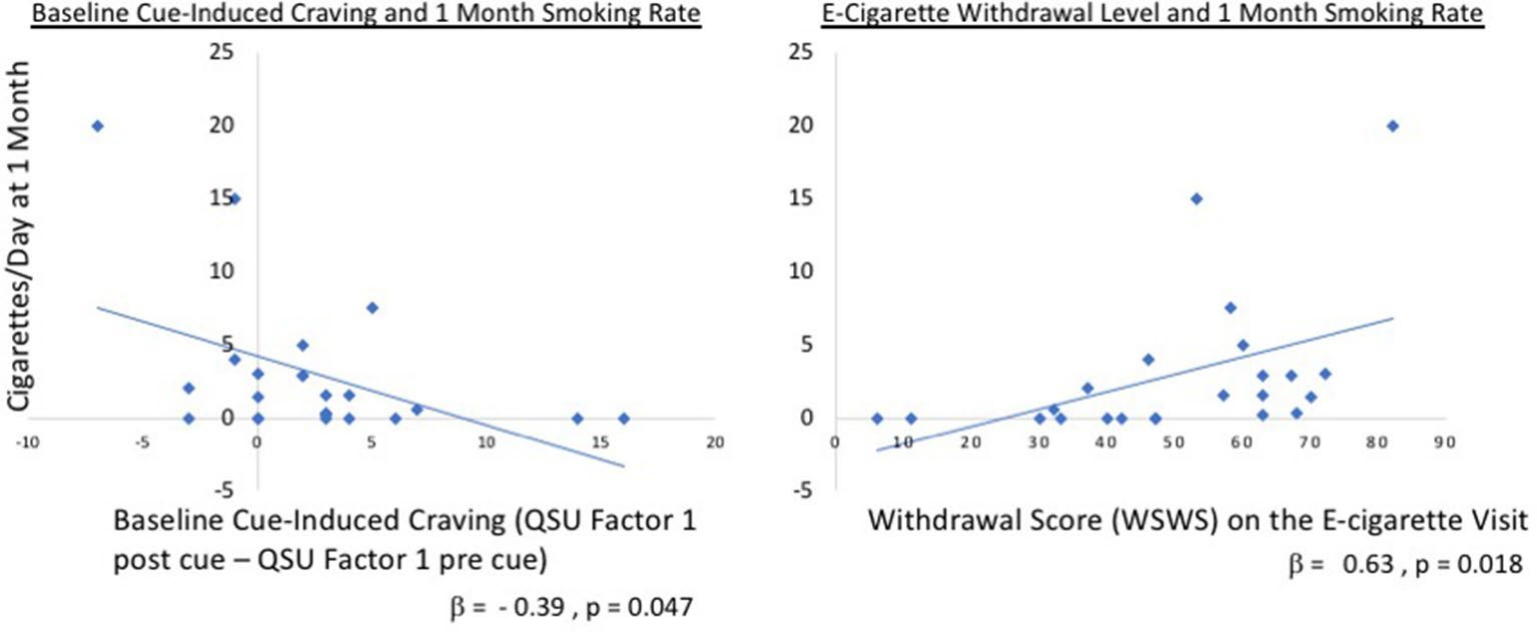
Regression tobacco cigarettes/day versus QSU factors. Relationship between cigarettes smoked at 1 month and cue-induced craving at baseline (left panel; Factor 1 QSU_post cue_ – Factor 1 QSU_pre cue_) and withdrawal on the e-cigarette visit (right panel). Overall, the model including cue-induced craving and WSWS at both the baseline and e-cigarette visits was significant *F*(4,17) = 3.82, *p* = 0.022 with an *R* *^2^* = 0.47. This was driven, in part, by the baseline difference in QSU factor 1 (standardized β = −0.39, *t* = −2.13, *p* = 0.047) and withdrawal on the e-cigarette visit (β = 0.630, *t* = 2.62, *p* = 0.018). QSU, Questionnaire of Smoking Urges, WSWS, Wisconsin Smoking Withdrawal Scale.

One month after completing the study, 34.8% of participants remained completely abstinent from smoking tobacco cigarettes. An additional 39% of individuals were smoking less than three cigarettes per day. A paired samples t-test revealed that participants continued to report significantly lower tobacco cigarette use at 1 month (*M* *_2_* = 2.94) compared to baseline [*M* *_1_* = 12.20; *t*(22) = 6.937, *p* < 0.001; **Figure 4**], confirmed by an almost significant reduction in breath CO [*M* *_1_* = 12.26, *M* *_2_* = 9.52; *t*(22) = 2.044, *p* = 0.053; **Figure 4**]. Accordingly, cigarette craving (without cue exposure) was significantly reduced at the follow-up (*M* *_2_* = 13.52) as compared to baseline [before cue exposure; *M* *_1_* = 17.83; *t*(22) = 3.963, *p* = 0.001].

### Predictors of 1-Month Cue Sensitivity and Cigarette Use

To determine whether cue-induced craving or withdrawal influenced the amount of tobacco smoking at 1-month follow-up, a linear regression was examined where number of cigarettes smoked per day was entered as the dependent variable. Baseline and e-cigarette values for WSWS and cue-induced QSU factor 1 differences (QSU_post cue exposure_ – QSU_pre cue exposure_) were included as predictors. Overall, the model was significant *F*(4,17) = 3.82, *p* = 0.022 with an *R* *^2^* = 0.47 (**Figure 4**), which was driven, in part, by the baseline difference in QSU factor 1 (standardized = −0.39, *t* = −2.13, *p* = 0.047) and withdrawal as measured on the e-cigarette visit (β = 0.630, *t* = 2.62, *p* = 0.018).

## Discussion

The present pilot study provides initial evidence that smoking cue reactivity is reduced when nicotine-free e-cigarettes are used in conjunction with transdermal NRT. Consistent with our prior study (19), our cue reactivity task induced significant amounts of craving at the baseline visit. In contrast, enhanced cue reactivity was not present after 2 weeks of nicotine-free e-cigarette and transdermal NRT use. These findings were specific to QSU factor 1, which supports the notion that the motivational aspects of the cues were devalued. In contrast, there was no change in QSU factor 2 measures, which was unaffected by cues and focuses on the motivation to mitigate withdrawal-related symptoms. This reduction in cue-induced craving is unlikely to be caused by NRT because there is ample evidence demonstrating that transdermal NRT does *not* mitigate cue-induced craving at the doses examined here and at higher doses (3, 5). This implies that, for some individuals who are less impacted by withdrawal symptoms, the use of nicotine-free e-cigarettes alone may be sufficient to aid in abstinence. However, for this initial investigation, we chose to address both cue reactivity and pharmacological withdrawal to reduce the potential for relapse caused by either factor. This design is limited in that the effect of NRT and e-cigarettes alone cannot be separated, and the independent effects of each intervention require further testing.

It is unlikely that habituation played a role because cue reactivity elicits a consistent response even across multiple visits (6, 14). It should be noted that craving before cue exposure was measured after smoking either a tobacco cigarette during the baseline visit or an e-cigarette during the second visit. The fact that the magnitude of craving did not differ after smoking either type of cigarette suggests that recent smoking has a similar impact on craving in this context, regardless of cigarette type or nicotine content.

One interesting discovery was that individuals who had the greatest baseline reactivity to smoking cues appeared to benefit the most from this combined intervention. Our prior work showed that, when using NRT alone, highly cue-reactive individuals were more likely to relapse (9), indicating that the addition of nicotine-free e-cigarettes likely aided cessation in this otherwise vulnerable population. However, withdrawal symptoms were significantly elevated during the e-cigarette visit relative to the baseline visit, and such withdrawal symptoms after the intervention were associated with more smoking at the 1-month follow-up visit. This suggests that a longer, more traditional course of NRT or an alternative pharmacotherapy may be more effective when paired with non-nicotine e-cigarettes. Future experiments should evaluate cessation aids, such as varenicline, and combined transdermal and short-acting NRT (e.g., lozenges, gum). It is plausible that such pharmacotherapies and nicotine-free e-cigarettes may impact cue reactivity via different mechanisms and thus in combination may enhance efficacy. These types of approaches are needed to confirm this hypothesis.

This pilot study supports the proposed proof of concept that non-nicotine-containing e-cigarettes and transdermal NRT reduce cue reactivity. For reasons previously discussed, this initial work focused only on those receiving both treatments, and next steps will aid in documenting the influence of both interventions. Another limitation of the current approach is that the e-cigarettes used cannot be quantified into distinct units, such as “whole cigarettes,” given that they do not burn down like combustible cigarettes. A single e-cigarette cartridge is equivalent to approximately one pack of cigarettes, but the length of time the cartridge lasts depends on how the individual uses it. For instance, one participant reported using the e-cigarette “continuously,” making it difficult to equate to a specific number of individual cigarettes. Should products be developed that allow for more fine-grained assessment of e-cigarette usage, this would aid in the understanding of how much use is needed to see the currently reported reduction in cue-induced craving. However, the present work shows that 2 weeks of *ad lib* use results in reduced cue reactivity, suggesting that allowing individuals to titrate use based on personal desire is effective. Furthermore, although NRT and e-cigarettes were not provided to participants after the 2-week intervention, it is possible that participants independently sought out and used these products in the interim leading up to the 1-month follow-up visit. However, on the 1-month visit, participants did not report using either NRT or e-cigarettes. Extended use of both NRT and e-cigarettes likely facilitates abstinence, and larger clinical trials are needed to determine how long of an intervention is needed to sustain longer-term abstinence. The current preliminary findings support the potential of such an approach.

These results are promising and suggest that providing patients with nicotine-free e-cigarettes along with NRT should continue to be explored as a combination strategy to reduce cue-induced craving and relapse vulnerability. Although safety trials involving nicotine-free e-cigarettes have not been conducted, clinical trials studying nicotine containing e-cigarettes have reported low adverse events related to their use (20, 21). In the aggregate, it is possible that non-nicotine-containing e-cigarettes could be developed as a harm reduction and/or cessation strategy for tobacco smokers wishing to quit.

## Data Availability

The datasets for this study will not be made publicly available because the work presented is preliminary and part of a larger on-going study and thus is still proprietary.

## Ethics Statement

All procedures were completed at McLean Hospital and the protocol was approved by the Partners Human Research Committee. Participants provided both verbal and written informed consent after receiving a complete description of the study.

## Author Contributions

AJ and SL designed the experiment. AP, EM, and AJ conducted data analysis. MZ collected the data. AP created the initial manuscript draft, which was finalized by AJ and SL with the insight and feedback from all authors.

## Funding

This research was funded by the National Institute of Drug Abuse (R01 DA039135, K02 DA042987 (ACJ)).

## Conflict of Interest Statement

The authors declare that the research was conducted in the absence of any commercial or financial relationships that could be construed as a potential conflict of interest.

The reviewer NC declared a shared affiliation, with no collaboration, with several of the authors AP, EM, SL, AJ to the handling editor.
